# Salivary Gland Scintigraphy in Sjögren's Syndrome: A Retrospective Study of Diagnostic Accuracy and Correlation With Histological and Immunological Biomarkers

**DOI:** 10.7759/cureus.65305

**Published:** 2024-07-24

**Authors:** Jorge Álvarez Troncoso, Luisa F Giraldo González, Mónica Coronado Poggio, Raquel Sorriguieta Torre, Elena Ruiz Bravo-Burguillos, Luis Domínguez Gadea, Clara Soto Abánades

**Affiliations:** 1 Internal Medicine, Hospital Universitario La Paz, Madrid, ESP; 2 Nuclear Medicine, Hospital Universitario La Paz, Madrid, ESP; 3 Pathology, Hospital Universitario La Paz, Madrid, ESP

**Keywords:** sjögren's syndrome, diagnostic performance, qualitative and quantitative research, whole unstimulated salivary flow, schirmer's test, anti-ro/ssa, biomarkers, minor salivary gland biopsy, scintigraphy, sicca syndrome

## Abstract

Introduction

Sicca syndrome, characterized by xerophthalmia and xerostomia, is associated with various autoimmune and non-autoimmune conditions, posing diagnostic challenges. Sjögren's syndrome (SS) is the most prevalent systemic autoimmune disease linked to sicca symptoms. This study evaluates the diagnostic accuracy of salivary gland scintigraphy (SGS) in distinguishing SS from non-Sjögren's sicca conditions, alongside other diagnostic tests.

Methods

A retrospective analysis was conducted at Hospital Universitario La Paz from December 2019 to March 2023, including 142 patients diagnosed with sicca syndrome. Correlations between qualitative and quantitative SGS data (GE Healthcare, Chicago, Illinois) and multiparametric sicca evaluations were assessed.

Results

Among the 142 patients, 84 (59.15%) were classified as having SS, with 55 (65.48%) seropositive for anti-Ro antibodies. Abnormal SGS results were found in 135 (95.07%) patients. Qualitative SGS categorized seven (4.93%) as mild, 53 (37.32%) as moderate, 50 (35.21%) as severe, and 21 (14.79%) as functionally annulled. Moderate or worse impairment had a sensitivity of 0.88 and a specificity of 0.17. Functional annulment had a sensitivity of 0.17 and a specificity of 0.97. Quantitative SGS using ejection fraction thresholds of ≤30% and ≤20% had sensitivities of 0.35 and 0.18 and specificities of 0.84 and 0.94, respectively. Quantitative SGS metrics correlated with unstimulated whole salivary flow (WUSF; 0.243; p=0.003) and inversely with lymphocytic infiltration (-0.281; p=0.001). The 2016 American College of Rheumatology/European League Against Rheumatism (ACR-EULAR) classification criteria for Sjögren's syndrome demonstrated an area under the curve (AUC) of 0.932, which improved to 0.951 with the inclusion of SGS parameters.

Conclusions

SGS is a significant diagnostic tool in the multiparametric evaluation of sicca syndrome, showing strong correlations with histological and immunological markers. Its integration into diagnostic criteria enhances the differentiation between SS and non-Sjögren's sicca conditions, suggesting its potential inclusion in future classification frameworks.

## Introduction

Sicca syndrome, characterized by ocular dryness (xerophthalmia) and oral dryness (xerostomia), is frequently associated with various autoimmune and non-autoimmune diseases [1]. This symptomatology presents a diagnostic challenge due to its presence across diverse disorders. Sjögren's syndrome (SS) is the most common systemic autoimmune disease associated with sicca symptoms [1, 2]. SS is a chronic inflammatory disease primarily targeting the exocrine glands, leading to their progressive degeneration and dysfunction [2].

The clinical landscape of SS is complex, with manifestations ranging from hallmark sicca symptoms to systemic involvement. Its diagnosis is often challenging due to the heterogeneous nature of clinical features and symptom overlap with other conditions [3]. The 2016 American College of Rheumatology/European League Against Rheumatism (ACR-EULAR) classification criteria for Sjögren's syndrome are pivotal in refining the diagnostic algorithm for SS. These criteria include 1) anti-Ro/SSA antibody positivity, 2) focal lymphocytic sialadenitis with a focus score ≥1 in minor salivary gland biopsy (MSGB), 3) ocular staining score (OSS) ≥5, 4) Schirmer's test ≤5 mm/5 min, and 5) whole unstimulated salivary flow (WUSF) ≤0.1 mL/min [4].

Objective diagnostic tests, such as the Schirmer test, WUSF, MSGB, and anti-Ro (SSA) antibody detection, are emphasized by these criteria [4,5]. The Schirmer test and OSS quantify tear production, which is essential for evaluating xerophthalmia. WUSF measures salivary gland functionality, while MSGB provides histopathological insights into glandular conditions. Anti-Ro antibody detection serves as a specific serological marker, significantly supporting SS diagnosis even in the absence of classic sicca symptoms [4,5].

However, these diagnostic tests have limitations and may not fully capture SS's complexity [3]. A multiparametric diagnostic approach is recommended for a comprehensive evaluation [3,5,6].

Salivary gland scintigraphy (SGS) is a crucial diagnostic imaging technique for assessing salivary gland function, particularly in SS. A study reported that SGS exhibited a sensitivity of 85.3% and a specificity of 77.8% [7]. This non-invasive nuclear medicine procedure evaluates gland function by monitoring technetium-99 uptake and secretion in the parotid and submandibular glands [8]. SGS involves both qualitative and quantitative analyses. Qualitative analysis requires visual scrutiny of scintigraphic images and time-activity curves, which can vary between interpreters. These curves exhibit patterns-normal, flat, or slow uptake and excretion-indicating the gland's functional status. Quantitative analysis provides objective evaluation by calculating parameters such as uptake ratio, excretion fraction, and maximum accumulation, offering precise quantification of gland function.

SGS correlates with salivary gland ultrasound [9] and histological findings [10], enhancing its diagnostic utility. Ultrasound imaging provides anatomical details that complement the functional data from SGS, while histological evaluations, often considered the gold standard for diagnosing salivary gland disorders, show high concordance with SGS results. This synergy between SGS, ultrasound, and histology provides a comprehensive diagnostic framework, allowing a nuanced understanding of salivary gland pathology in systemic autoimmune diseases, particularly SS [11]. Recent research suggests that SGS can also monitor disease progression and treatment efficacy in SS [12].

This study aimed to investigate the diagnostic accuracy of SGS in a multiparametric evaluation to differentiate SS from non-Sjögren sicca conditions. We conducted a thorough analysis of qualitative and quantitative SGS data, correlating them with WUSF, Schirmer's test, MSGB findings, and specific immunological markers such as the antinuclear antibody (ANA), anti-Ro, anti-La, and rheumatoid factor. Our integrative approach seeks to establish SGS as a cornerstone in the multidisciplinary diagnostic framework for SS, improving diagnostic accuracy and therapeutic management by guiding the use of pilocarpine and other secretagogues.

## Materials and methods

Study design and population

**Figure 1:**
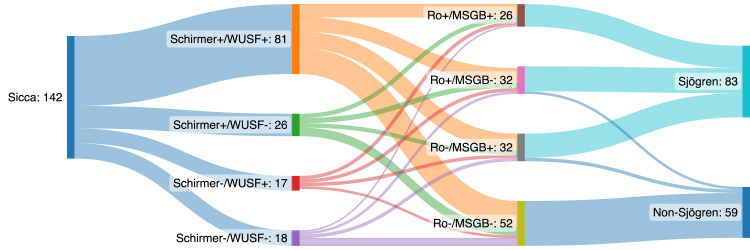
Sankey diagram showing patient flow throughout the different tests in the multiparametric evaluation Sankey diagram displaying the progression of 142 patients in this study through multiparametric evaluation for sicca syndrome. Starting with all patients, the diagram branches according to the results of the Schirmer and WUSF tests, then further divides based on anti-Ro antibody presence and MSGB results. Finally, it shows the distribution of patients diagnosed with either Sjögren's syndrome or non-Sjögren's syndrome, with the width of the bands representing the number of patients in each pathway. WUSF - whole unstimulated saliva flow; MSGB - minor salivary gland biopsy

We conducted a retrospective, unicentric study from December 2019 to March 2023 at the Systemic Autoimmune Diseases Unit of Hospital Universitario La Paz. The study included medical records of consecutive patients diagnosed with Sicca syndrome (Figure 1). The primary objective was to elucidate the correlation between Sicca syndrome and parameters included in the 2016 ACR-EULAR classification criteria for Sjögren's syndrome. Medical records were stored in the hospital's electronic health record system and were accessed by authorized personnel for the purpose of this study. The study was approved by the Ethics Commission of the Hospital Universitario La Paz (PI-5756) and complied with the Declaration of Helsinki.

Assessments of classification criteria

Demographic, clinical, histological, and immunological parameters were meticulously evaluated. The diagnosis of SS adhered to the 2016 ACR-EULAR classification criteria for Sjögren's syndrome, requiring a minimum score of four from the weighted criteria.

Salivary gland scintigraphy (SGS)

**Figure 2:**
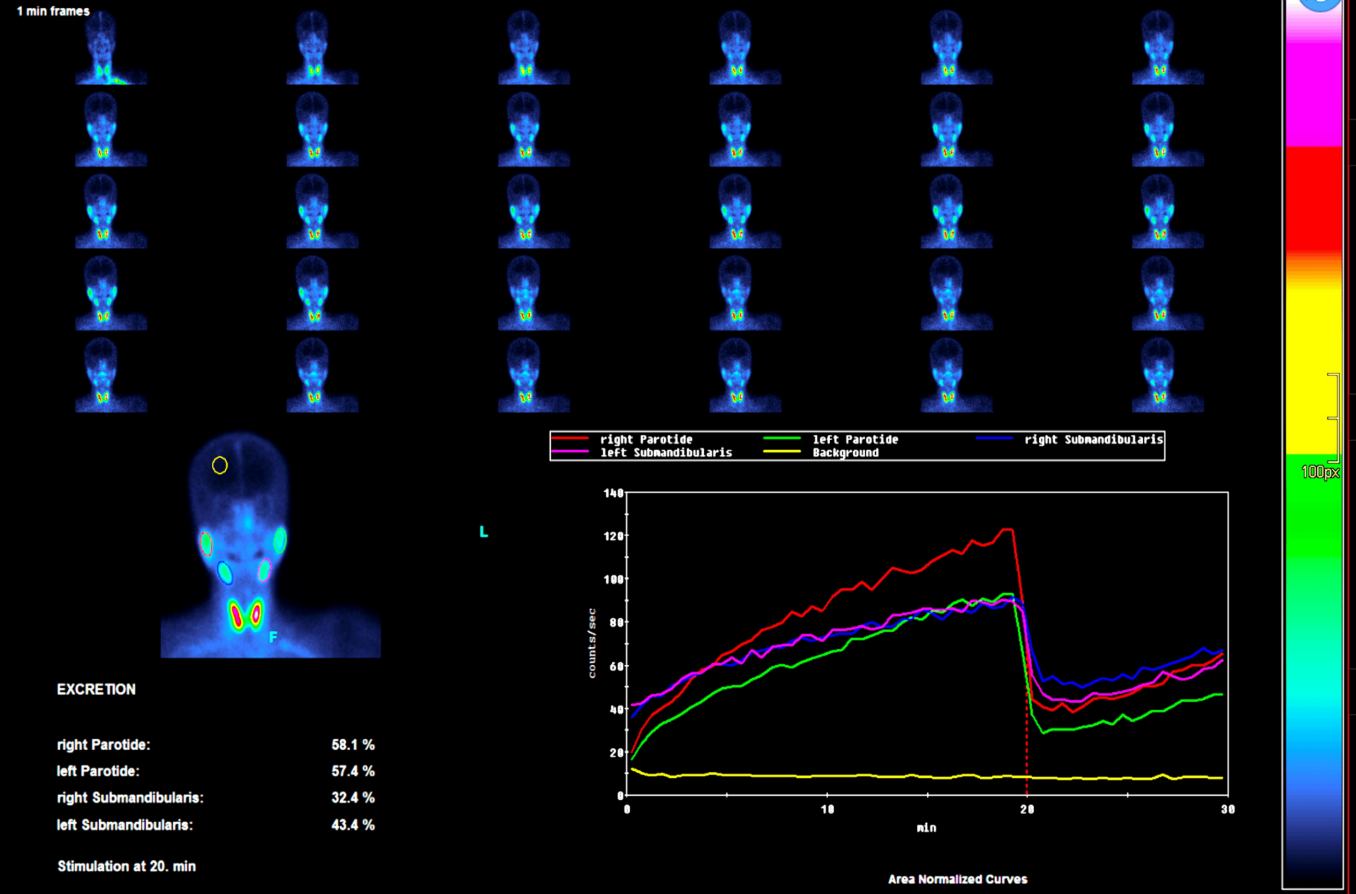
Salivary gland scintigraphy of a patient with Sjögren's syndrome with qualitative and quantitative measures The left side shows sequential scintigraphy images over time, indicating the uptake of a radioactive tracer. The single image below shows tracer distribution in the parotid and submandibular glands, with excretion percentages listed, showing how much tracer is expelled post-stimulation. On the right, a graph with time-activity curves for each gland demonstrates the uptake and subsequent excretion of the tracer, with the drop after 20 minutes marking stimulation. This figure illustrates the diminished function of the salivary glands, which is characteristic of Sjögren's syndrome.

Patients fasted for two to six hours before SGS. They were positioned supine on the imaging table, and imaging was performed using the GE Discovery™ 670 DR gamma camera (GE Healthcare, Chicago, Illinois), with its anterior head aligned over the patient's head. A dynamic study of 60 images was acquired at 30-second intervals, beginning from the moment of radiopharmaceutical intravenous injection of 185 Mbq of 99mTc-pertechnetate. Lemon juice was administered at the 20-minute mark to stimulate salivary secretion. Study processing was performed on a Xeleris workstation (GE Healthcare, Chicago, Illinois). Sequential images were presented using a French scale color. The uptake ratio was calculated by dividing the mean counts of each region of interest (ROI) by the background. The excretion fraction was calculated as (counts in the salivary gland at 20 min - counts in the salivary gland after sialogogue administration) / (counts in the salivary gland at 20 min) x 100%. Activity/time curves for each gland were obtained. A five-scoring visual system (Schall's classification) defined salivary gland uptake as normal (similar to the thyroid), mildly decreased, moderately decreased, severely decreased, and functional annulment (uptake similar to the background; Figure 2). This standardized protocol aimed to ensure the reliability and reproducibility of SGS, accurately quantifying stimulated flow rates and assessing the functional reserve of salivary glands for well-informed treatment planning.

Statistical analysis

Descriptive statistics summarized clinical and demographic data. Categorical variables were reported as frequencies and percentages, while continuous variables were presented as means and standard deviations (SD) for normally distributed data or medians and interquartile ranges (IQR) for non-normally distributed data. Concordance between two expert SGS readers was assessed using the kappa coefficient. The Chi-squared test analyzed categorical variables. Logistic regression models identified variables influencing treatment response, controlling for potential confounders or effect modifiers. Confidence intervals were estimated using robust methods. Multivariate logistic regression incorporated variables with p-values <0.1 from the univariate analysis, along with other potential influencers of treatment response. Statistical significance was established at p-values <0.05. Analyses were performed using R version 4.3.1 for Windows (R Foundation, Vienna, Austria) and Wizard Pro for Mac version 2.0.12.

## Results

Demographic data, clinical characteristics, and serological profile

The study evaluated 142 patients diagnosed with Sicca syndrome (Figure 1), of whom 127 (89.44%) were women and 122 (85.92%) were of Spanish descent (Table 1). The participants had an average age of 56.08 years (±1.46). The mean 2016 ACR-EULAR classification criteria score was 3.89 (±2.33), and 84 (59.15%) were classified as having Sjögren's syndrome (SS). Within this subset, 55 (65.48%) were seropositive for anti-Ro antibodies, while 29 (34.52%) were seronegative. Serological evaluations indicated that 92 (64.79%) of patients tested positive for antinuclear antibodies (ANA), 60 (42.25%) for anti-extractable nuclear antigen (anti-ENA), 58 (40.85%) for anti-Ro, 16 (11.27%) for anti-La/SSB, and 31 (21.83%) for rheumatoid factor (RF). Conversely, 44 (30.99%) showed negative results for ANA, anti-Ro, anti-La, and RF. The causes of sicca in non-Sjögren patients were mainly drug-related (72.41%). However, these causes were not specifically calculated or detailed in the manuscript as it was not the primary objective of the study.

**Table 1:** Demographics, clinical characteristics, and serological profile ANA - antinuclear antibody; ENA - extractable nuclear antigen; WUSF - whole unstimulated salivary flow; FS - focus score

Characteristic	Sjögren's (n=84, 59.15%)	Non-Sjögren's (n=58, 40.85%)	p-value
Female sex	78 (92.86%)	49 (84.48%)	0.111
Age ≥50 years	59 (70.24%)	42 (72.41%)	0.779
ANA positivity	70 (83.33%)	22 (37.93%)	<0.001
ENA positivity	55 (65.48%)	5 (8.62%)	<0.001
Anti-Ro positivity	55 (65.48%)	3 (5.17%)	<0.001
Anti-La positivity	16 (19.05%)	0 (0.00%)	<0.001
Rheumatoid factor positivity	26 (30.95%)	5 (8.62%)	0.002
Schirmer positivity	68 (80.95%)	39 (67.24%)	0.062
WUSF positivity	66 (78.57%)	32 (55.17%)	0.003
FS ≥1	40 (47.62%)	3 (5.17%)	<0.001
Focal lymphocytic sialadenitis	53 (63.10%)	6 (10.34%)	<0.001

Multiparametric assessment

Abnormal results were observed in 124 (87.32%) of the patients using either the Schirmer test or the whole unstimulated salivary flow (WUSF) test. The mean Schirmer test result was 4.39 mm (±5.68), with 107 (75.35%) tests indicating abnormal results (≤5 mm/5 min). The mean WUSF was 0.13 ml/min (±0.19), with 98 (69.01%) tests yielding abnormal results (≤0.1 ml/min). Minor salivary gland biopsy (MSGB) revealed abnormal findings in 129 (90.85%) patients. Among these, 59 (41.55%) had focal lymphocytic sialadenitis. Only 43 (30.28%) had a focus score (FS) of ≥1, meeting the diagnostic criteria for SS. The mean FS was 0.90 (±1.43).

Comparative analysis: Sjögren's vs. non-Sjögren's cohorts

No significant differences were observed between SS patients and non-Sjögren's patients in terms of gender or age. However, the SS cohort showed significantly elevated rates of ANA, ENA, anti-Ro, anti-La, and RF positivity. Furthermore, the SS group had a higher rate of WUSF positivity (78.57% vs. 55.17%, p=0.003) and FS≥1 (47.62% vs. 5.17%, p<0.001), but not in the Schirmer test positivity. The SS group also exhibited a higher prevalence of focal lymphocytic sialadenitis (63.10% vs. 10.34%, p<0.001).

Salivary gland scintigraphy (SGS) outcomes

Abnormal SGS results were identified in 135 (95.07%) of the patients. The qualitative evaluation (Table 2) categorized seven (4.93%) as mild, 53 (37.32%) as moderate, 50 (35.21%) as severe, and 21 (14.79%) as functionally annulled. The kappa coefficient agreement was 0.76 (p=0.005). Table 2 outlines the SGS results for distinguishing SS from non-Sjögren's sicca conditions. It reveals that both groups generally have similar SGS results, except for the functional annulment and abnormal time-activity curve categories, where SS patients show higher frequencies, suggesting more severe gland dysfunction. Quantitatively, while uptake ratios are comparable across both conditions, significant differences emerge at specific ejection fraction (EF) cutoff points (≤30% and ≤20%), indicating that SS patients have markedly reduced salivary gland function. These findings underscore SGS's potential in identifying SS by highlighting functional impairments that are not as evident in non-Sjögren's sicca cases.

**Table 2:** Salivary gland scintigraphy (SGS) outcomes

Salivary gland scintigraphy (SGS) outcomes	Sjögren's (n=84, 59.15%)	Non-Sjögren's (n=58, 40.85%)	p-value
1. Qualitative assessment			
Normal	4 (4.76%)	3 (5.17%)	0.912
Mild	4 (4.76%)	7 (12.07%)	0.109
Moderate	30 (35.71%)	23 (39.65%)	0.633
Severe	28 (33.33%)	22 (37.93%)	0.573
Functional annulment	18 (21.43%)	3 (5.17%)	0.007
2. Semiquantitative assessment			
Abnormal time-activity curve	16 (19.05%)	4 (6.90%)	0.041
3. Quantitative assessment			
3.1. Uptake ratio (UR)			
Mean uptake ratio at parotid glands (cps/s)	110.24	119.08	0.730
Mean uptake ratio at submandibular glands (cps/s)	106.20	120.73	0.156
Mean global uptake ratio (cps/s)	108.22	119.91	0.385
3.2. Ejection fraction (EF)			
Mean ejection fraction at parotid glands (%)	37.92	42.87	0.452
Mean ejection fraction at submandibular glands (%)	29.37	33.10	0.120
Mean global ejection fraction (%)	33.64	37.99	0.220

Diagnostic tools evaluation

The study evaluated the diagnostic accuracy of various tests for distinguishing Sjögren's Syndrome from non-Sjögren's sicca. The qualitative abnormal SGS showed a sensitivity of 0.98 and a specificity of 0.05. Adjusting the threshold to moderate or worse impairment, the sensitivity was 0.88 with a specificity of 0.17. For functional annulment in qualitative SGS, the sensitivity was 0.17, and the specificity was 0.97.

Quantitative SGS with an EF threshold of ≤30% had a sensitivity of 0.35 and a specificity of 0.84. An EF threshold of <20% yielded a sensitivity of 0.18 and a specificity of 0.94. MSGB with an FS≥1 showed a sensitivity of 0.48 and a specificity of 0.95. The Schirmer test and WUSF demonstrated sensitivities of 0.81 and 0.79 and specificities of 0.33 and 0.45, respectively. Detection of anti-Ro antibodies presented a sensitivity of 0.65 and a specificity of 0.95, as detailed in Table 3, informing the differentiation between SS and non-Sjögren's sicca.

**Table 3:** Diagnostic performance of tests in differentiating Sjögren's syndrome from non-Sjögren's sicca SGS - salivary gland scintigraphy; EF - ejection fraction; MSGB - minor salivary gland biopsy; FS - focus score; WUSF - whole unstimulated salivary flow

Test	Sensitivity	Specificity	Positive predictive value	Negative predictive value
Qualitative SGS				
Abnormal	0.98	0.05	0.60	0.60
Moderate or worse	0.88	0.17	0.61	0.50
Functional annulment	0.17	0.97	0.88	0.44
Semiquantitative SGS				
Time-activity curve	0.19	0.93	0.80	0.44
Quantitative SGS				
EF ≤30%	0.35	0.84	0.74	0.47
EF ≤20%	0.18	0.94	0.94	0.45
Multiparametric evaluation				
MSGB with FS ≥1	0.48	0.95	0.93	0.56
Schirmer's test	0.81	0.33	0.64	0.54
WUSF	0.79	0.45	0.67	0.59
Anti-Ro/SSA	0.65	0.95	0.95	0.65

Correlations between SGS, histological and immunological parameters

**Figure 3:**
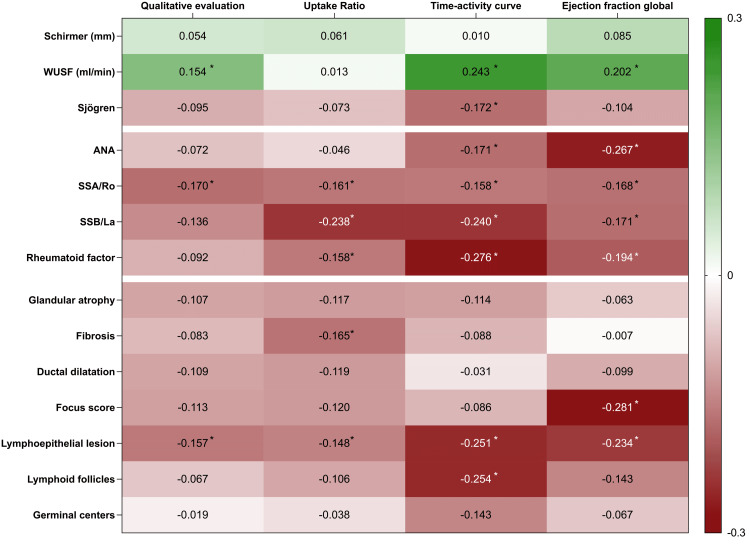
Correlation coefficients and statistical significance of SGS parameters with immunological markers and histological features in sicca evaluation Heat-map correlation matrix reveals several statistically significant relationships, indicated by asterisks, between SGS parameters and specific immunological and histological features in sicca syndrome. The color coding ranges from green (positive correlation) to red (negative correlation), representing the strength and direction of each relationship. Darker shades signify stronger correlations ANA - antinuclear antibody

Within the scope of the SGS results, histological correlations were evident. Lymphoepithelial lesions were notably correlated with the functional annulment of SGS (qualitative evaluation), as reflected by a correlation coefficient of -0.157 and a significant p-value (p=0.002), as shown in Figure 3. The EF showed a correlation with WUSF (0.243; p=0.003) and decreased with increased lymphocytic infiltration (-0.281; p=0.001). Histological characteristics such as glandular atrophy, fibrosis, and ductal dilation were not significantly correlated with SGS metrics.

Immunological data indicated that the presence of specific autoantibodies correlated with SGS results. ANA, anti-Ro/SSA, anti-La/SSB, and rheumatoid factor showed significant associations with pathological SGS findings and reduced ejection fractions. These correlations were more pronounced with an increasing number of positive autoantibodies detected.

In our cohort, the 2016 ACR-EULAR Classification Criteria for Sjögren's Syndrome demonstrated an area under the curve (AUC) of 0.932 for diagnostic performance. This metric was further enhanced to an AUC of 0.951 after integrating the time-activity curve and EF from SGS into the diagnostic evaluation.

## Discussion

Our investigation assessed the diagnostic accuracy of SGS for distinguishing SS from non-Sjögren's sicca conditions. Our data highlight the value of SGS as a complement to the diagnostic toolkit for SS, providing detailed information on salivary gland function not readily obtained from other tests [2,3,11].

The literature supports the application of SGS in evaluating sicca syndrome, and our findings are consistent with these reports [7-13]. SGS is critical for assessing glandular dysfunction in SS, a disease characterized by exocrine gland impairment [1-5]. SGS offers a non-invasive means to evaluate the pathophysiological trajectory of SS, with studies suggesting its utility in both diagnosis and disease monitoring, potentially improving patient care [11,13,14]. Although not part of the ACR-EULAR classification criteria [4], our results suggest that SGS, through comprehensive qualitative, semiquantitative, and quantitative evaluation, could bridge diagnostic gaps [4,5].

Qualitative SGS analysis enables visual evaluation of glandular function. When combined with semiquantitative and quantitative measurements, it provides a robust method to establish specific diagnostic thresholds. For example, our study found that a mean global EF≤20% was highly specific for SS, suggesting its use as a potential diagnostic marker.

SGS findings correlated strongly with histological and immunological parameters, reinforcing their role in understanding SS's complex immunopathology [9-14]. These correlations underscore the importance of SGS as a potential diagnostic tool despite the condition's clinical heterogeneity.

However, the utility of SGS should not overshadow the need for a comprehensive diagnostic approach that includes clinical, laboratory, and additional imaging data, such as ultrasound, for a definitive diagnosis of SS. Future research should aim to refine SS diagnostic criteria and explore new modalities that can enhance existing protocols [3,5,6].

The strength of this study lies in the holistic evaluation of sicca syndrome, shedding light on SS's pathogenesis. The study's limitations include its retrospective design, limited sample size, and single-center scope, which may affect generalizability. Drug-related causes of non-Sjögren sicca were noted but not specifically analyzed, and differentiation between Ro52 and Ro60 reactivity was not made. Additionally, SGS is not part of the current ACR-EULAR classification criteria. Despite these limitations, SGS demonstrated significant diagnostic value and should be used alongside other clinical, laboratory, and imaging assessments. Prospective studies with broader cohorts are needed to confirm our findings. Further investigation could explore the prognostic capabilities of SGS in SS progression [13].

In conclusion, SGS has shown significant diagnostic value in this study, underscoring its importance in differentiating Sicca syndrome and SS. By enriching the multiparametric diagnostic approach, SGS contributes to refining diagnoses and improving the precision of clinical assessments [2,3,11].

## Conclusions

In our study, we investigated the diagnostic utility of SGS within a comprehensive multiparametric framework for distinguishing SS from non-Sjögren's sicca conditions. By incorporating qualitative and quantitative SGS data with established clinical assessments, we identified significant correlations between SGS outcomes and specific histological and immunological markers, enhancing diagnostic accuracy for SS and improving the AUC.

Although SGS is not a standalone diagnostic tool, it complements a holistic diagnostic approach that includes clinical, laboratory, and imaging evaluations. The findings suggest future research should further validate the role of SGS in diagnosing and monitoring SS. The integration of SGS parameters augments diagnostic accuracy, supporting its potential inclusion in future classification criteria for more comprehensive disease assessment.
